# Production of engineered long-life and male sterile *Pelargonium* plants

**DOI:** 10.1186/1471-2229-12-156

**Published:** 2012-08-31

**Authors:** Begoña García-Sogo, Benito Pineda, Edelín Roque, Teresa Antón, Alejandro Atarés, Marisé Borja, José Pío Beltrán, Vicente Moreno, Luis Antonio Cañas

**Affiliations:** 1 Instituto de Biología Molecular y Celular de Plantas (CSIC-UPV), Ciudad Politécnica de la Innovación, Edf. 8E. C/Ingeniero Fausto Elio s/n, Valencia E-46011, Spain; 2 BIOMIVA S.L, Carretera M-511 Km. 2, Villaviciosa de Odón Madrid, E-28670, Spain; 3 Plant Response Biotech S.L. Parque Científico-Tecnológico Montegancedo, Pozuelo de Alarcón, Madrid, E-28223, Spain

**Keywords:** *Pelargonium zonale*, *Pelargonium peltatum*, *pSAG12* promoter, *ipt* gene, Engineered long-lived plants, Delayed senescence, Engineered male sterility, *PsEND1* promoter, Barnase, Anther ablation, Biosafe ornamentals

## Abstract

**Background:**

Pelargonium is one of the most popular garden plants in the world. Moreover, it has a considerable economic importance in the ornamental plant market. Conventional cross-breeding strategies have generated a range of cultivars with excellent traits. However, gene transfer via *Agrobacterium tumefaciens* could be a helpful tool to further improve Pelargonium by enabling the introduction of new genes/traits*.* We report a simple and reliable protocol for the genetic transformation of *Pelargonium spp.* and the production of engineered long-life and male sterile *Pelargonium zonale* plants, using the *pSAG12::ipt* and *PsEND1::barnase* chimaeric genes respectively.

**Results:**

The *pSAG12::ipt* transgenic plants showed delayed leaf senescence, increased branching and reduced internodal length, as compared to control plants. Leaves and flowers of the *pSAG12::ipt* plants were reduced in size and displayed a more intense coloration. In the transgenic lines carrying the *PsEND1::barnase* construct no pollen grains were observed in the modified anther structures, which developed instead of normal anthers. The locules of sterile anthers collapsed 3–4 days prior to floral anthesis and, in most cases, the undeveloped anther tissues underwent necrosis.

**Conclusion:**

The chimaeric construct *pSAG12::ipt* can be useful in *Pelargonium* spp. to delay the senescence process and to modify plant architecture. In addition, the use of engineered male sterile plants would be especially useful to produce environmentally friendly transgenic plants carrying new traits by preventing gene flow between the genetically modified ornamentals and related plant species. These characteristics could be of interest, from a commercial point of view, both for pelargonium producers and consumers.

## Background

The genus Pelargonium (family Geraniaceae), with more than 200 species, represents one of the most popular garden plants around the world, having a considerable economic importance in the market of ornamental plants. The vegetative-propagated bedding plants from the two groups *Pelargonium zonale* (syn. *Pelargonium* x *hortorum*) and *Pelargonium peltatum* hybrids are the most cultivated potted plants. In addition, species of Pelargonium are important in the perfume industry and are cultivated and distilled for their scent.

*Pelargonium spp.* grow as annuals in temperate climates. Leaves are usually alternate and palmately lobed or pinnate, often on long stalks and sometimes with light or dark patterns. The erect stems bear five-petaled zygomorphic flowers in umbel-like clusters (pseudo umbels). These plants have been bred to produce a variety of flower shapes, ranging from star-shaped to funnel-shaped, and colors, such as white, pink, red, orange-red, fuchsia or deep purple. Pelargonium breeding programmers have yielded new flower colors and shapes, early and continuous flowering, good postharvest and market quality and pest and disease resistance.

Gene transfer by means of *Agrobacterium tumefaciens* enables the introduction of new genes from unrelated species and would be a helpful tool for further progress in pelargonium breeding. The first step for applying genetic transformation via *Agrobacterium* is to develop an efficient *in vitro* regeneration system for the target plant. It is also beneficial to develop methods to propagate explants from adult plants of genotypes tested for agronomic performance.

The vegetative-propagated pelargoniums are highly heterozygous resulting in variable offspring [1]. For *P. zonale*, as well as *P. peltatum* hybrids, several reports have been published dealing with *in vitro* regeneration from explants taken from adult plants, using either organogenesis [2-7] or somatic embryogenesis [5,7-11]. *Agrobacterium*-mediated transformation of different pelargonium genotypes was reported by several authors using different marker and reporter genes [1,4,9,12-14], however no reports on the use of the *green fluorescent protein* (*gfp*) gene as an *in vivo* selectable marker have been published. G*fp* expression in transformed cells should be useful to identify transformation events at early stages, such that selectable marker genes (antibiotic or herbicide resistance) may not be required.

Cytokinins have been implicated in several aspects of plant development, including plant senescence [15-20], and are thought to be synthesized mainly in the roots and transported to the shoots via the xylem. Concentrations of endogenous cytokinins decline in plant tissues as senescence progresses. The cytokinin content of the xylem sap of sunflower (*Helianthus annuus*) and soybean (*Glycine max*) also decreases rapidly with the onset of senescence, which suggests that reduction in cytokinin transport from roots to shoots allows senescence to progress [21,22]. Moreover, exogenous application of cytokinins has been exploited commercially to extend the shelf life of freshly harvested vegetables and cut flowers [23].

Transgene-encoded cytokinin biosynthesis was initially studied in *Nicotiana tabacum* using constitutive or inducible overexpression of the *isopentenyl phosphotransferase* (*ipt*) gene of *Agrobacterium tumefaciens*. This enzyme catalyzes the rate-limiting step for *de novo* cytokinin biosynthesis in plants [24]. Isopentenyl AMP is the precursor of all other cytokinins, of which the three most commonly detected and physiologically active forms are isopentenyl adenine (IPA), zeatin (Z), and dihydrozeatin (DHZ) [25]. Overexpression of the *ipt* gene in transgenic plants led to elevated foliar cytokinin concentrations and delayed leaf senescence, but high cytokinin levels have been reported to be detrimental to growth and fertility [26-30]. To circumvent these effects, Gan and Amasino [31] devised a strategy, based on auto-regulated cytokinin production, which delayed leaf senescence in transgenic tobacco without altering other plant phenotypes. This strategy exploited a senescence-specific gene promoter (*pSAG12*) from an *Arabidopsis thaliana* gene [32], fused to the *ipt* gene (*tmr* gene from the Ti plasmid of *A. tumefaciens*) [33]. The *pSAG12::ipt* chimaeric gene was reported to be activated only at the onset of senescence in the lower mature leaves of tobacco. This approach resulted in cytokinin biosynthesis restricted to the leaves, which inhibited leaf senescence, preventing cytokinin overproduction. The ability to delay leaf senescence has potential for crop improvement. The effect of *pSAG12*::*ipt* expression in transgenic plants has been assessed in several Solanaceous crops [17], and in rice (*Oryza sativa*) [34], cauliflower (*Brassica oleracea*) [35] and lettuce (*Lactuca sativa* L. cv. Evola) [36,37]. In potted ornamental plants, like pelargoniums, a delay in the senescence process would be of interest both for consumers and producers.

Engineered male sterility in ornamental plants has many applications such as hybrid seed production, elimination of pollen allergens, reduction of the need for deadheading to extend the flowering period, redirection of resources from seeds to vegetative growth and increase of flower longevity. The use of this technology could be especially useful to produce environmentally friendly transgenic ornamentals carrying new traits, as this modification would prevent gene flow between the genetically modified plants and related species [38-40]. Expression of the *barnase* gene under control of the anther-specific *PsEND1* promoter [38,39,41,42] may be used to efficiently create male sterile versions of existing pelargonium cultivars without adversely affecting the respective phenotypes. The *PsEND1* promoter shows specific expression in those tissues involved in anther architecture of many plant species. *PsEND1* is a pea anther-specific gene that displays very early expression in the anther primordium cells. Later on, *PsEND1* expression becomes restricted to the epidermis, connective, endothecium and middle layer, but it is never observed in the anther filament, tapetal cells or microsporocytes. The expression pattern of this gene continues until floral anthesis. In addition, the expression of *barstar*, an inhibitor of the ribonuclease barnase, has been used to restore fertility to plants with barnase-induced sterility [43,44] and to prevent the possible effects of ectopic *barnase* expression in engineered male and female sterile plants [40].

The main objectives of this work were to develop a simple and reliable *Agrobacterium*-mediated protocol for the genetic transformation of *Pelargonium zonale* and *P. peltatum* using the *gfp* gene as an *in vivo* marker, to test the effects of *pSAG12::ipt* expression on leaf senescence and plant morphology, and to engineer male sterility in this ornamental crop by ablating tissues essential for the anther development and subsequently for pollen growth using the *PsEND1::barnase* chimaeric gene. The potential commercial applications (increased shelf life, reduced plant architecture) of transgene-encoded auto-regulated cytokinin biosynthesis in this ornamental plant and the production of male sterile lines to prevent undesirable gene flow between the genetically modified plants and related species are also discussed.

## Methods

### Plant material and tissue culture

**Figure 1:**
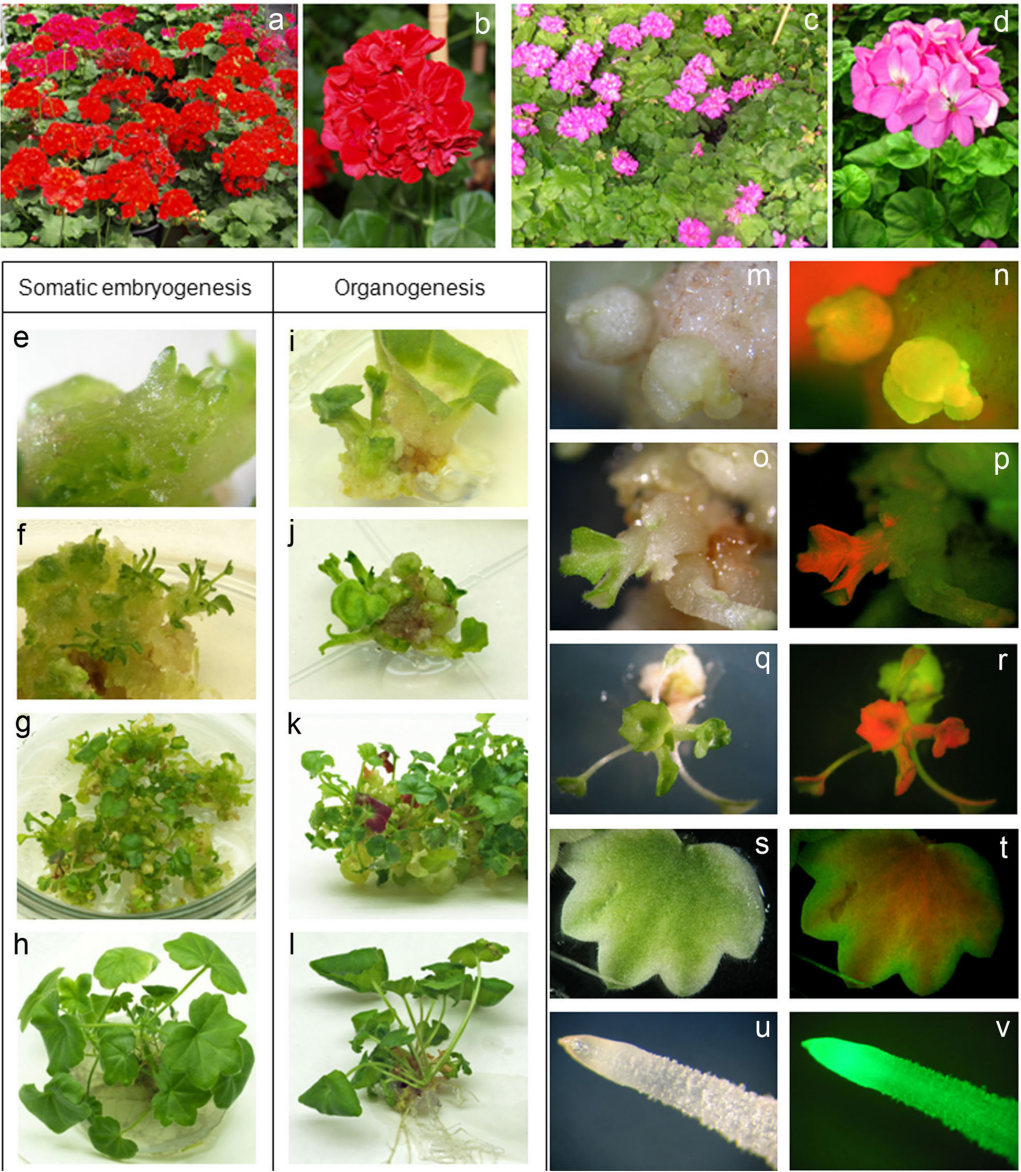
***In vitro *regeneration of transgenic plants via somatic embryogenesis in *Pelargonium peltatum *and via organogenesis in *P. zonale*. (a) ***P. peltatum *WT plants. **(b) ***P. peltatum *inflorescence. **(c) ***P. zonale *WT plants. **(d) ***P. zonale *inflorescence. **(e-f)**. Production of somatic embryos in a callus of *P. peltatum *cultivated in selective Morphogenesis Induction Medium (MIM). **(g)**. *P. peltatum *developing embryos in selective Elongation Medium (EM). **(h)**. *P. peltatum* transgenic plantlets in Rooting Medium (RM). **(i-j)**. Adventitious buds in a callus of *P. zonale *in selective MIM. **(k)**. *P. zonale *shoot elongation in selective EM. **(l)**. *P. zonale *rooted plantlet in RM. **(m-n)**. Detection of transformation events in both *Pelargonium *spp., GFP green fluorescence is clearly visible in the initial whitish callus with a disorganized growth. **(o-p)**. Chlorophyll shows strong red autofluorescence that could mask the green fluorescence of transformed cells, it becomes increasingly difficult to identify in the subsequent organogenic callus and in the adventitious buds. **(q-r)**. Green fluorescence was observed in regenerated shoots but is masked by the chlorophyll in the young leaves. **(s-t)**. Green fluorescence can be observed in the periphery of young leaves where chlorophyll does not accumulate. **(u-v)**. Green fluorescence is especially evident in the roots, where the chlorophyll is absent. In general, within the same organ, GFP detection varied in different tissues or cell types depending on their chlorophyll content.

*Pelargonium peltatum* cv. Aranjuez (Figure 1a-b) and *Pelargonium zonale* (syn. *Pelargonium* x *hortorum*) cv. 370 (Figure 1c-d) *in vitro* propagated plants were used as source of explants. Young leaf explants (first to the fifth leaf from the apex) were harvested from 30–40 days old axenically grown plantlets propagated from shoot segments. Axenic plants were established by surface sterilization of 2 cm shoot segments processing an axillary bud excised from greenhouse grown plants. These nodal cuttings were first washed thoroughly with water, and then surface-sterilized by immersion in a 2.5% solution of sodium hypochlorite with 0.1% of 7X-O-matic detergent (Flow Laboratories) for 20 min and rinsed three times with sterile distilled water. Once sterilized, the nodal cuttings were cultured on Rooting Medium (RM) composed of Murashige and Skoog (MS) basal medium [45], 20 mg l^-1^ sucrose, 1 mg l^-1^ thiamine-HCl, 100 mg l^-1^ myo-inositol, 8 g l^-1^ agar and 0.1 mg l^-1^ IAA. Axenic plants obtained from shoots segments were propagated every two months in 580 ml culture vessels with 60 ml of RM and maintained as *in vitro* stock plants. All cultures were incubated at 25°C under a 16 h photoperiod with fluorescent light (60 μmol m^-2^ s^-1^ intensity).

*In vitro* plant regeneration was carried out using a Morphogenesis Induction Medium (MIM) composed of MS basal medium and Shahin [46] vitamins supplemented with 30 g l^-1^ sucrose, 100 mg l^-1^ myo-inositol, 8 g l^-1^ agar, IAA (0.01 mg l^-1^), TDZ (0,5 mg l^-1^) and 1 mg l^-1^ of Cu-sulphate. The pH was adjusted to 5.7 before autoclaving. The petiole proximal area of axenic leaves was cut into 1 cm^2^ pieces and cultured on MIM with the abaxial surface in contact with the medium. Regeneration in *Pelargonium zonale* was carried out via direct organogenesis and in *Pelargonium peltatum* via somatic embryogenesis.

### *A. tumefaciens* strain and chimaeric gene constructs

*Agrobacterium* strain LBA4404 was used in all transformation experiments. LBA4404 cells were electroporated to carry different plasmids: i) a pBIN19 binary vector harboring, from the right to the left border, the *nptII* marker gene under the control of the *nos* promoter and the *nos* terminator, and the *gfp-S65T*[47] reporter gene under the control of a 2X *35SCaMV* constitutive promoter and the *nos* terminator; ii) a plasmid derived from pBI101 harboring, from the right to the left border, the *nptII* marker gene under the control of the *nos* promoter and the *nos* terminator, and the *barnase* gene under the control of the *PsEND1* promoter and the *nos* terminator (pBI101-*PsEND1*::*barnase* construct) [38], and iii) a plasmid (pVDH393) carrying the *ipt* gene under the control of the senescence inducible *pSAG12* promoter and with the *nos* terminator, the reporter gene *uidA* (GUS-intron) under the control of the *35SCaMV* promoter and with the *35SCaMV* terminator and the *nptII* marker gene under the control of the *35SCaMV* promoter and with the *35SCaMV* terminator (pVDH393-*pSAG12*::*ipt* construct; supplied by company Van der Have, ND). Bacteria were grown at 28°C on solid LB plates supplemented with 40 mg l^-1^ rifampicin and 100 mg l^-1^ kanamycin. A single colony was used to inoculate 25 ml of LB liquid medium with the same antibiotics. Flasks were maintained at 28°C and 200 rpm for 24 h and later on were used to inoculate a liquid MS medium supplemented with 20 g l^-1^ sucrose, 100 mg l^-1^ myo-inositol, 1 mg l^-1^ thiamine-HCl, 100 mg l^-1^ 2-(*N*-morpholino)ethane sulfonic acid (MES) and 0.2 mM acetosyringone dissolved in 70% ethanol (sterilized by filtration), which was cultured at 28°C for 12 h. Inoculation of explants was conducted when the bacterial culture reached an OD (600 nm) of 0.06.

The promoter region of the *PsEND1* gene (GenBank accession n.: AY324651) was previously cloned into the binary vector pBI101 where the −2736/-6 promoter fragment was fused to the coding sequence of the β-glucuronidase (*uidA*) reporter gene [41] PsEND1*::barnase-barstar* chimaeric gene: primers Ribo1 (5’-*TAGGATCCC*GACCATGGCACAGGTTATC-3’) and Inhi2 (5´-*GCGAGCTC*TTAAGAAAGTTGATGGTGATG-3´) were designed based on the published sequence of *barnase* and *barstar* genes [48] to amplify the *barnase-barstar* fragment and to introduce *Bam*HI and *Sac*I restriction sites. Barnase is a very active ribonuclease. Even a low level of expression from aberrant promoter sequences or run-off expression from neighboring genes during manipulations in *E. coli* or *Agrobacterium* would have prevented the survival of the bacteria. Therefore, the *barstar* gene which encodes an inhibitor of barnase is included in the construct. The PCR resulting fragment was cloned into the pGEM-T Easy (Promega) and later released with *the Bam*HI and *Sac*I enzymes. The *Bam*HI*-Sac*I fragment was cloned by replacement of the *uidA* coding sequence into the binary vector pBI101 generating the pBI101-*PsEND1::barnase-barstar* construct. The *nos::ntpII* plant selectable marker gene, which confers resistance to kanamycin in transgenic plants, was also introduced in the T-DNA.

### Transformation and regeneration of transgenic plants

Transformation experiments were carried out using both *Pelargonium zonale* and *Pelargonium peltatum* leaf explants as starting material*.* Leaf explants were prepared from two months old axenic plants as described above. Explants were inoculated in groups of 25 explants with 50 ml of the bacterial suspension for 5 min and thereafter transferred to co-culture medium consisting of MIM supplemented with 0.2 mM acetosyringone. Explants were co-cultured for 2–3 days in the dark at 25°C, afterwards they were transferred into sterile glass jars containing a liquid washing medium (MS basal medium, 20 mg l^-1^ sucrose, 1 mg l^-1^ thiamine-HCl, 100 mg l^-1^ myo-inositol, 100 mg l^-1^ MES and 600 mg l^-1^ cefotaxime) and soaked for 5 min. The explants were subsequently blotted dry with sterile filter paper and subculture onto selective MIM supplemented with 300 mg l^-1^ timentin and 50 mg l^-1^ kanamycin for *Agrobacterium* eradication and selection of transgenic events, respectively. All antibiotics were filter sterilized and added to cooled media (45°C) before pouring into 9 cm diameter Petri dishes as 25 ml medium per plate. Control explants were treated in the same manner, except for the inoculation with *Agrobacterium*. Control groups were established and cultured on medium with and without kanamycin. All explants were subculture every 2 weeks onto the same fresh medium until shoots were long enough to be separated from the callus. After 2.5-3 months in culture, calli showing well developed morphogenetic structures (shoots in the case of *P. zonale* and somatic embryos in *P. peltatum*) were transferred to a selective Elongation Medium (EM: MS basal medium and Shahin vitamins, supplemented with 30 g l^-1^ sucrose, 100 mg l^-1^ myo-inositol, 8 g l^-1^ agar, 0.01 mg l^-1^ NAA, 0.1 mg l^-1^ 6 BA, 1 mg l^-1^ of Cu-sulphate, 300 mg l^-1^ timentin and 50 mg l^-1^ kanamycin). After 1–1.5 months in EM, the shoots were cut and cultivated in Rooting Medium (RM). Regenerated plantlets with well-developed roots were transferred to plastic pots containing peat moss and perlite (3:1) as substrate and acclimatized in growth chambers initially covered with a transparent plastic to maintain humidity. Plants were cultivated under long day conditions (16-h light/8-h dark photoperiod) and then transferred to a greenhouse until they flowered. Transformation efficiency was estimated as the number of independent transformation events (one transgenic plant per explant) in relation to the total number of inoculated explants.

### Ploidy level analysis

*Pelargonium zonale* and *P. peltatum* have 2n = 18 chromosomes. Modern cultivars have been obtained by intra- and inter-specific hybridization and usually they show high ploidy levels when compared with wild species. The ploidy level was evaluated by flow cytometry as described previously [49]. Leaf tissue from *in vitro* plants was used for nuclei isolation. Pieces of tissue (1 cm^2^) were chopped individually on a glass plate with a sharp razor blade in 200 μl of nuclei isolation buffer (Partec). The sample was then passed through a 50 μm nylon filter and 800 μl of staining solution (Partec), containing 1 mg l^-1^ DAPI (4,6-diamino-2-phenyl-indole), were added for DNA fluorescence. The DNA content of the isolated nuclei was measured using a Partec PAS-II flow cytometer equipped with a mercury lamp. Fluorophore excitation peak is below 420 nm and fluorescence emission peak for DAPI is between 435 and 500 nm. The data were plotted on a histogram where the horizontal axis shows DNA content (proportional to fluorescence) and the vertical axis shows nuclei number. About 5000 to 10000 nuclei were measured per sample. Analyses were carried with young leaves from adult plants of the original cultivar and young leaves from transgenic plants.

### PCR analysis

Plant DNA used for PCR analysis was extracted from young leaves using the protocol of Rogers and Bendich [50]. PCR analysis was carried out for all transgene using the following primer pairs: for the *nptII* gene, forward primer KAN-1: (5’-AAG ATG GAT TGC ACG CAG GTT C-3’) and reverse primer KAN-2: (5’-GAA GAA CTC GTC AAG AAG GCG A-3’); for the *uidA* gene, forward primer GUS-1: (5’-ATC AGG AAG TGA TGG AGC ATC A-3’) and reverse primer GUS-2: (5’GGT GAT CGG ACG CGT CGG GTC G-3’); for the *gfp* gene, forward primer GFP-D: (5’-ATG GTG AGC CAA GGG CGA GGA-3’) and reverse primer GFP-R: (5’-GGA CCA TGT GAT CGC GCT TC-3)’; for the *barnase-barstar* genes, forward primer Ribo3 (5´-ACG GAC CAT TAT CAG ACC TTT AC-3´) and reverse primer Inhi3 (5´-CGC AGC CTT CCG CTT TCG C-3´); for *ipt* gene, forward primer IPTDIR (5’-GGT CCA ACT TGC ACA GGA AAG-3’ and reverse primer IPTREV: (5’-CCC TCC AAA GTT GAA CCA ACT C-3’). The PCR reactions were carried out in a total volume of 20 μl comprising 0.1-0.2 μg genomic DNA, 0.2 mM of each dNTPs, 1.5 mM MgCl_2_, 0.6 μM 5’ and 3’ primers and 0.5 U Taq DNA polymerase. For *nptII* analysis, DNA was denatured at 94°C for 5 min followed by 30 cycles (94°C for 30 s, 56°C for 45 s, 72°C for 1 min) and finally 10 min at 72°C. For *uidA* analysis, DNA was denatured at 94°C for 5 min followed by 30 cycles of (94°C for 30 s, 60°C for 45 s, 72°C for 1 min) and finally 10 min at 72°C. For *gfp* analysis, DNA was denatured at 94°C for 5 min followed by 35 cycles of (94°C for 30 s, 60°C for 30s, 72°C for 1 min) and finally 10 min at 72°C. For *barnase-barstar* and *ipt* analyses, DNA was denatured at 94°C for 5 min followed by 35 cycles of (94°C for 30 s, 55°C for 30s, 72°C for 1 min) and finally 10 min at 72°C. The expected products sizes were 781 bp for *nptII*, 1021 bp for *uidA*, 661 bp for *gpf*, 544 bp for *barnase-barstar* and 460 bp for *ipt* gene. PCR products were detected by UV light after electrophoresis on 1% w/v agarose ethidium bromide gels.

### Real-time RT-PCR analysis

Total RNA was isolated from detached leaves of the *P. zonale* transgenic lines 3.4, 3.9, 4.3, 4.12 and from leaves of WT control plants using Plant RNA Purification Reagent protocol (Invitrogen Corporation, Carlsbad, CA). DNase treatment of the RNA preparations for real-time reverse transcription-polymerase chain reaction (Real-Time RT-PCR) was performed using the Turbo DNA-Free Kit (Ambion, Austin, TX) according to the manufacturer’s specifications. RNA concentration for each sample was measured spectrophotometrically (NanoDropTechnologies, Inc., Wilmington, DE) and the quality was visually assessed by formaldehyde-agarose gel electrophoresis. For first-strand synthesis, total RNA (1 μg) was reverse-transcribed in a 20 μl reaction mixture using the PrimerScript 1^st^ strand cDNA Synthesis Kit (TAKARA; http.//http://www.takara-bio.co.jp). Two microliters of RT reaction were used for a Real-Time RT-PCR analysis with 300 nM of each primer mixed with the Power SYBR ® Green PCR Master Mix (Applied Biosystems) as indicated manufacturer’s instructions. The reactions were carried out into 96 weel-optical reaction plates using ABI PRISM 7500 Sequence Detection System and appropriated software (Applied Biosystems). The data were normalized using *Pelargonium x hortorum PhACTIN7*[51]*.* Relative expression values were calculated after normalizing against the maximum expression value. Primers were designed for all genes using PRIMER EXPRESS version 2.0 (Applied Biosystems) with default parameters. Primers used were as follow: *ipt* (isopentenyl transferase): ipt-qRT-DIR: 5’-GCTACCCAGAACCAGATCACG-3’ and Ipt-qRT-REV: 5’-ATCTGCGTCGAGCTGCAATA-3’ and *PhACTIN7*: PhACTIN7-qRT-DIR: 5’-TCCAGCAGATGTGGATTTCAAA-3’ and PhACT7-qRT-REV: 5’-TTGATGGGCCAGACTCATCAT-3’.

Analysis of gene expression for each sample was performed on three experimental repeats with real-time RT-PCR for both *ipt* and *PhACTIN7*. The actual value of each pair (*ipt- PhACTIN7*) is equal to 2^-ΔΔCt^. Each sample’s expression level relative to *PhACTIN7* is the mean of three biological repeats. The relative expression level of the sample with the highest expression level was set to one and all other samples values were normalized to that value to generate the fold.

### Light microscopy

Transformed explants were examined periodically for *gfp* expression under a fluorescence stereomicroscope (Leica MZ FLIII) equipped with a Leica Fluorescence Module GFP3 comprising a 470–440 nm Excitation Filter and a 525–550 nm Barrier Filter. A mercury lamp provided the light source. The red autofluorescence from chlorophyll was not blocked with any filter. Floral buds and stamens from both transgenic and WT plants were freshly harvested and dissected using forceps and scalpel. Light photographs of dissected flowers and stamens were obtained using a stereomicroscope (MZ8, Leica).

β-glucuronidase activity was determined histochemically according to Jefferson et al. [52]. Root, shoot and leaf segments from the putative transgenic plants were stained for 24 h at 37°C, cleared with 70% ethanol and observed under a stereomicroscope (MZ8 Leica).

### Morphological measurements

Morphological measurements of vegetative growth were made to determine whether the *barnase* and *ipt* expression could affect different plant growth parameters. Measurements were taken in the greenhouse on T1 hemizygous transgenic plants and WT control plants. Plant height in flowering plants (distance from soil line to top of the tallest growing point), leaf length and width (average measurements from five fully expanded leaves), leaf petiole length, internodal length and number of inflorescences per plant were evaluated. Morphological measurements were taken over the course of several days on each plant as its first five flowers reached anthesis. Means differing significantly were compared using the Student test at a 5% probability level. Data variability was expressed as the mean ± SE.

### Quantification of chlorophyll

Analysis of leaf senescence was conducted by extraction of chlorophyll in detached leaves incubated in darkness from WT control and *pSAG12::ipt* plants respectively. Using a porcelain mortar cooled with liquid nitrogen, samples were crushed to a fine powder. In 10 ml centrifuge tubes the samples were mixed with 100 mg of MgCO_3_ and 5 ml of 100% (v/v) acetone. Bleached leaf material was removed by centrifugation (5 min; 2,000 *g*) and 1 ml aliquots of supernatants transferred to new tubes. Chlorophyll (a + b) content of extracts was determined spectrophotometrically [53].

## Results

### *In vitro* regeneration from leaf explants of axenic *P. peltatum* and *P. zonale* plants and selection with kanamycin

To induce *in vitro* plant regeneration, leaf explants were taken from axenically-propagated plants and cultured in Morphogenesis Induction Medium (MIM). By using axenic *Pelargonium peltatum* and *Pelargonium zonale* plants as the source for explants, the percentage of regenerating explants reached 90% in *P. peltatum* and 80% in *P. zonale*. Plant regeneration was carried out via somatic embryogenesis in *P. peltatum* (Figure 1e-h) and via organogenesis in *P. zonale* (Figure 1i-l).

Since the plasmids used for transformation experiments carried the *nptII* gene as the selectable marker, it was necessary to determine the suitable concentration of kanamycin for the selection of transgenic events. Leaf explants from axenic plants were cultured in selective MIM with different concentrations of kanamycin (0, 25, 50 and 100 mg l^-1^). A concentration of 50 mg l^-1^ was sufficient to inhibit the growth of non-transformed cells in leaf explants of both cultivars. Additionally, regenerated shoots were cultured in selective Elongation Medium (EM) with different kanamycin concentrations (0, 25, 50 and 100 mg l^-1^). According to our results (data not shown), a kanamycin concentration of 50 mg l^-1^ was used in EM to select transgenic plantlets for both cultivars.

### Genetic transformation experiments

Genetic transformation experiments of *P. zonale* and *P. peltatum* were conducted using leaf explants from axenic plants. In the initial transformation experiments, explants from both *Pelargonium spp*. were inoculated with *Agrobacterium tumefaciens* strain LBA4404 carrying the pBIN19 binary vector harbouring, between the left and right border, the *nptII* marker gene under the control of the *nos* promoter and the *nos* terminator and the *gfp-S65T* reporter gene under the control of a 2x35SCaMV constitutive promoter and the *nos* terminator. The explants were cultured in selective MIM with 50 mg l^-1^ kanamycin. After one month of culture, explants began to develop morphogenetic calli on the cut surface of the leaf explants. Non-transformed calli became necrotic after 30–50 days in selective medium. After 2.5-3 months in selective MIM, the calli differentiated adventitious shoots in the case of *P. zonale* and somatic embryos in *P. peltatum*, which were excised and cultured in selective EM. In some cases, several independent transformation events were identified within the same explant (adventitious shoots on opposite sides of the same explant), but only one plant per explant was recovered to be sure that all the selected plants were derived from independent transformation events. After 1–1.5 moths of culture in selective EM, the shoots were excised and cultured in Rooting Medium (RM). Finally, 6 independent rooted plants of *P. zonale* and 6 of *P. peltatum* were selected and transferred to pots. Transgenic plants were acclimatized in a growth chamber and transferred to the greenhouse, where they subsequently flowered normally under long day conditions. Transformation efficiency, estimated as the number of independent transformation events (one transgenic plant per explant) in relation to the total number of inoculated explants, ranged between 2-3% in both cultivars.

To produce long-life and male sterile pelargonium plants, we used the same transformation protocol described above and axenic leaves of *P. zonale* as the starting material*.* The leaf explants were co-cultivated with *A. tumefaciens* strain LBA4404 carrying the chimaeric constructs pVDH393-*pSAG12*::*ipt* and pBI101-*PsEND1*::*barnase* respectively.

### GFP as an *in vivo* selectable marker

While the *nptII* gene was employed for the selection of transgenic plants, the *gfp* gene expression was also examined during the transformation process in order to evaluate the ability of *gfp* as *in vivo* selectable marker. The expression of the *gfp* gene in the leaf explants was observed 40–50 days after inoculation (when adventitious buds or somatic embryos begin to appear) and also in the transgenic plants obtained. The *gfp* expression in transformed cells should be useful to select transformation events at early stages, so that antibiotic selection marker genes may not be required. However, in the case of *Pelargonium spp.*, if the selection based only on *gfp* expression, a significant number of transgenic plants could be undetected due to the presence of chlorophyll in the green tissues. This problem is related to the fluorescence visualization in tissues with high chlorophyll content, as in the case of leaves. Chlorophyll shows strong red autofluorescence that could mask the green fluorescence of a few cells. In both *Pelargonium spp*., green fluorescence is clearly visible in the initial whitish callus with disorganized growth (Figure 1m-n), while it becomes increasingly difficult to identify in the subsequent organogenic callus and in the adventitious buds (Figure 1o-p). All kanamycin resistant plants showed green fluorescence in different organs and tissues. Green fluorescence was observed in regenerated shoots and plantlets (Figure 1q-r), on the border of leaves (Figure 1s-t) and especially in the roots, where the chlorophyll is absent (Figure 1u-v). In general, within the same organ, *gfp* detection varied in different tissues or cell types depending on their chlorophyll content.

### Ploidy level analysis

**Figure 2:**
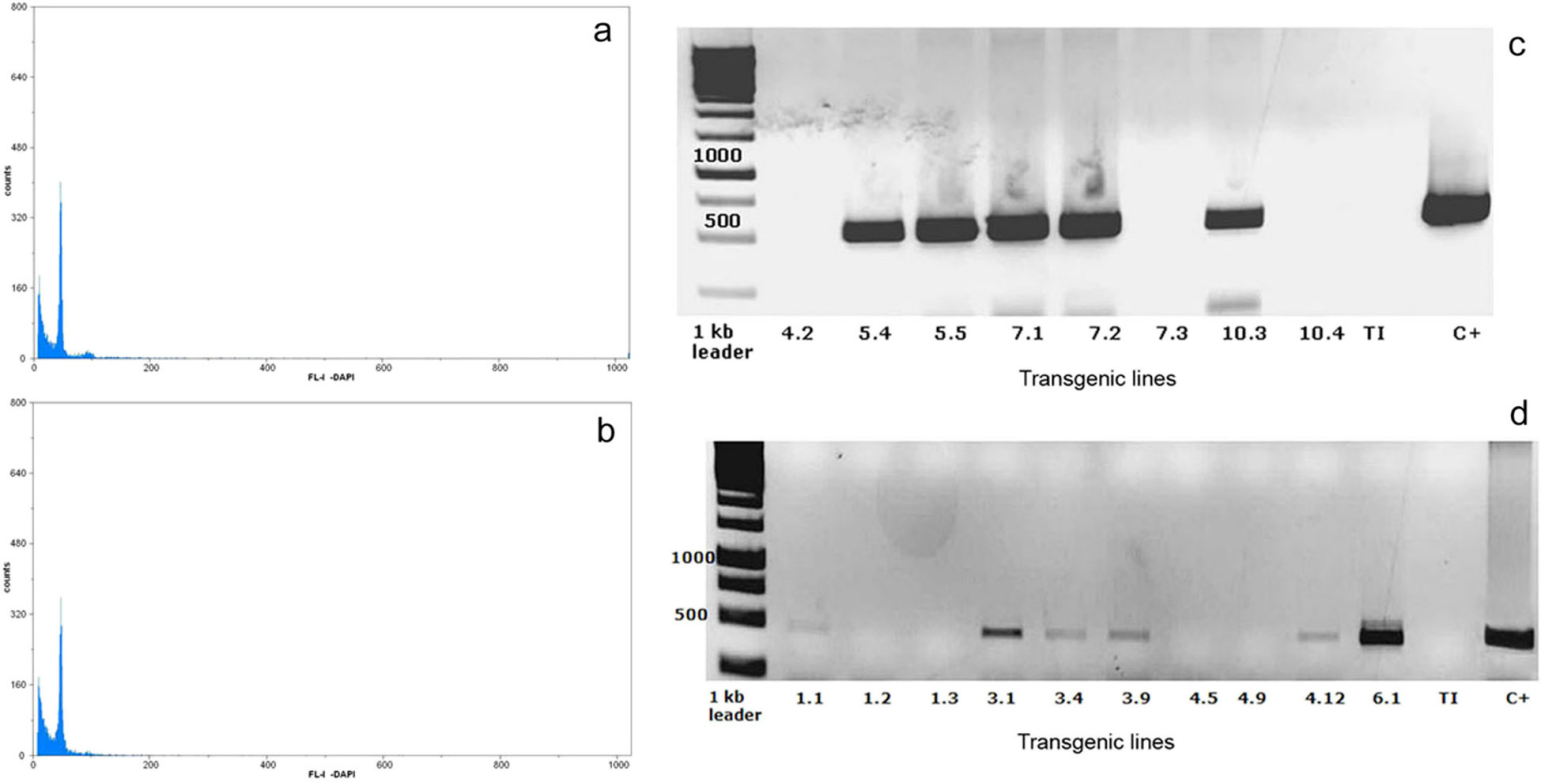
**Ploidy level and PCR analyses of transgenic pelargonium plants. **Histograms of number of nuclei per channel as a function of relative fluorescence intensity resulting from flow cytometry analysis of nuclei stained with DAPI. Nuclei were isolated from leaf samples of diploid WT control (**a**) and transgenic (**b**) plants. (**c**). Identification of the *barnase-barstar * transgene (544 bp fragment) by PCR in different *P. zonale *male sterile plants. C + (positive control: pBI101-*PsEND1*::*barnase-barstar*) and TI (negative control). (**d**). Identification of the *ipt *transgene (460 bp fragment) by PCR in different *P. zonale pSAG12::ipt *transgenic plants. C + (positive control: pVDH393-*pSAG12*::*ipt*) and TI (negative control).

The ploidy level of plantlets regenerated in selective medium was analyzed and the data were compared with those of the original materials. Analyses were carried out with control leaves from adult plants of the original cultivar and young leaves from transgenic plants (Figure 2a-b). Only diploid plants showing identical ploidy level as the original ones were selected.

### PCR analysis of the transgenes and selection of transgenic lines

The presence of the *nptII*, *uid*A, *gfp*, *barnase-barstar* and *ipt* transgenes in the selected transgenic lines of both *P. zonale* and *P. peltatum* was confirmed by PCR. Figure 2 shows the detection by PCR analysis of the *barnase-barstar* (Figure 2c) and *ipt* transgenes (Figure 2d) in different transgenic plants of *P. zonale.*

Based on the PCR analysis, we selected 12 independent lines of *P. zonale,* 6 carrying the pVDH393-*pSAG12::ipt* construct and 6 with the pBI101-*PsEND1::barnase* chimaeric gene for further phenotypic and molecular analyses.

**Figure 3:**
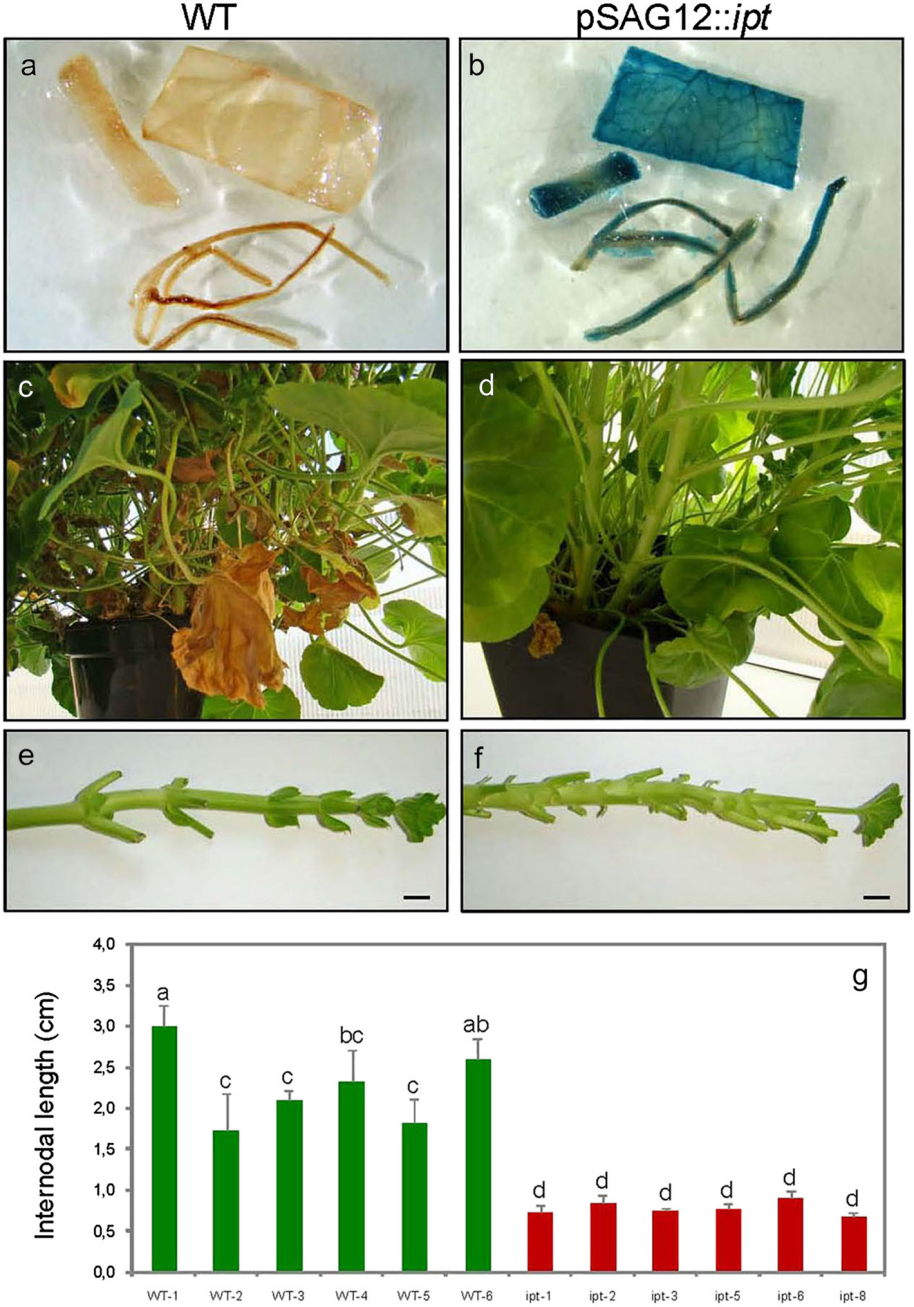
**Reduced internode length and increased branching in *Pelargonium zonale pSAG12::ipt *transgenic plants. **(**a-b**). Constitutive GUS expression in leaf, stem and root fragments in the *pSAG12::ipt *transgenic plants as additional proof of their genetic transformation. (**c-d**). Non-transformed WT potted plant (left) compared with a *pSAG12::ipt * transgenic plant (right). The transgenic plant showed delayed leaf senescence, more evident in the basal leaves and increased branching. (**e-f**). The *pSAG12::ipt *stems showed reduced internodal length (right) when compared with control WT plants. (**g**). Statistical analysis of the important differences in internodal length among 6 WT plants and 6 transgenic lines. The transgenic *pSAG12::ipt *plants showed a more compact architecture that the WT phenotype. Bars: 1 cm.

The selected transgenic lines of *P. zonale* carrying the *pSAG12::ipt* construct were also evaluated for GUS production, because they all carry the *uidA* reporter gene under the control of the *35SCaMV* promoter. In Figure 3a-b, constitutive GUS expression can be observed in stems, leaves and roots of the *pSAG12::ipt* plants in comparison with control plants (WT).

### Morphological measurements in selected transgenic lines

Morphological measurements of vegetative growth were taken over the course of several days for each plant as its first five flowers reached anthesis in order to determine whether transgene expression could affect different growth parameters. The measured parameters included plant height at flowering, leaf width, leaf length, leaf petiole length, node number, internodal length and number of inflorescences per plant. Our results indicate that the vegetative growth and flowering of the transgenic plants were not modified in a significant manner by *barnase* expression in the anthers (not shown). Therefore, it seems that there is no ectopic effect of the *barnase* gene in vegetative or reproductive plant tissues other than anthers, corroborating the tissue-specificity of the *PsEND1* promoter. In contrast, the transgenic *pSAG12::ipt* plants showed delayed leaf senescence, which is more evident in the basal leaves (Figure 3c-d), increased branching and shorter internodes than the WT, producing a phenotype that displays a more compact architecture of the whole plant (Figure 3e-f).

### Delayed senescence in *P. zonale* pVDH393-*pSAG12::ipt* transgenic plants

After rooting, the *in vitro* transgenic plants were acclimatized and cultivated in the greenhouse to evaluate their possible delayed senescence. Transgenic plants showed delayed leaf senescence, increased branching and reduced internodal length when compared with control WT plants (not transformed). Figure 3g shows the important differences in internodal length among 6 WT plants and 6 transgenic lines. The transgenic *pSAG12::ipt* plants showed a phenotype with more compact architecture that the WT phenotype.

**Figure 4:**
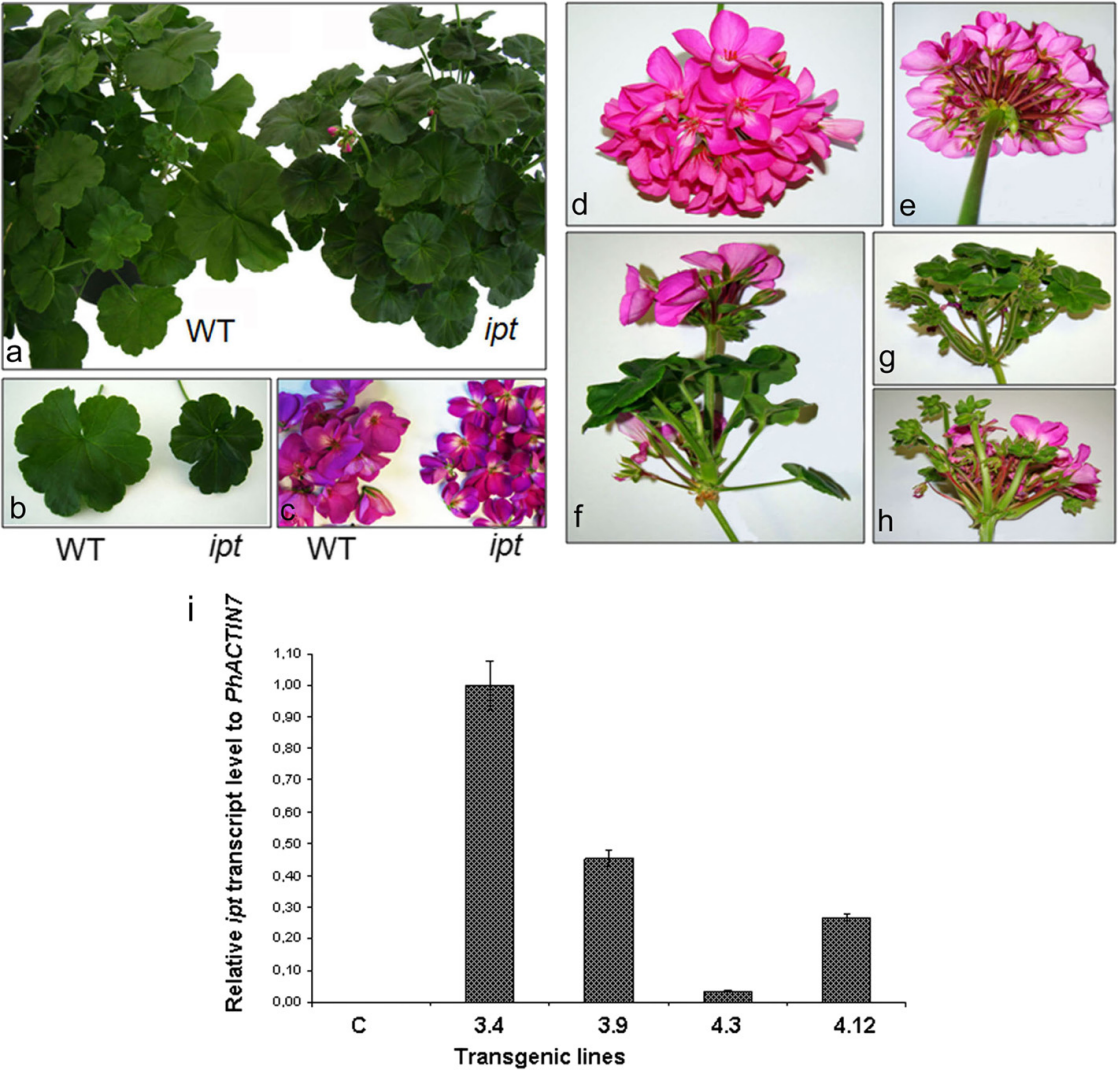
**Reduced leaf and flower size in *Pelargonium zonale pSAG12::ipt *transgenic plants. **Occasional inflorescence reversion. (**a**). The *pSAG12::ipt *transgenic plants (right) showed a more compact architecture with reduced organs than the WT plants (left). (**b**). Reduced leaf size in the *pSAG12::ipt *plants (right) compared with a WT leaf (left). (**c**). The flowers of the *pSAG12::ipt *plants were also reduced in size (right) when compared with WT flowers (left). In adult plants cultivated at the greenhouse, we did not observe alterations in the inflorescence development when compared the WT inflorescences with the transgenic ones (**d-e**). Occasionally, in some *pSAG12::ipt *inflorescences, flowers coexist with new vegetative structures, which are produced at the same time as flowers or new inflorescences, indicating inflorescence reversion (**f-g-h**). (**i**). Real-time RT-PCR analysis of *pSAG12::ipt *transcript levels in detached leaves from the transgenic lines 3.4, 3.9, 4.3 and 4.12. Each sample’s expression level relative to *Pelargonium x hortorum PhACTIN7 *is the mean of three biological repeats. C: control WT leaves.

Another phenotypic difference between the WT and the transgenic plants was the smaller leaf size observed in the *pSAG12::ipt* plants (Figure 4a-b). In the inflorescences, the flower phenotypes were quite similar, although the *pSAG12::ipt* flowers were also reduced in size when compared with WT flowers (Figure 4c). Transgenic leaves and flowers showed a more intense color than the non-transformed ones, probably as a consequence of their reduced size. In some transgenic adult plants, we occasionally observed alterations in the inflorescence development when comparing transgenic with WT inflorescences (Figure 4d-e). In some *pSAG12::ipt* inflorescences, flowers coexist with new vegetative structures, which are produced at the same time as flowers or new inflorescences (Figure 4f-g-h). This occasional phenomenon could be due to a large number of copies or the insertion position of the *pSAG12::ipt* transgene in the plant genome. To elucidate if there was a correlation between the observed phenotype and the expression level of the exogenous gene in these plants, we carried out real-time RT-PCR experiments with four selected transgenic lines showing delayed senescence (3.4, 3.9, 4.3 and 4.12). The transgenic line 3.4 showed an inflorescence reversion phenotype. Our results indicated that the expression level of the transgene is higher in line 3.4 when compared with the other ones (Figure 4i). However, this phenotype could be considered as an undesirable collateral effect from a commercial point of view, for this reason, transgenic lines showing occasional inflorescence reversion were discarded.

All of *P. zonale pSAG12::ipt* transgenic plants cultivated in the greenhouse exhibited delayed senescence when compared with WT control plants, especially at the basal leaves. In Figure 3, a comparison between adult basal leaves (more than 5 months) from *pSAG12::ipt* transgenic plants (Figure 3d) and basal leaves of non-transformed plants with a similar age (Figure 3c) was showed. A high number of adult leaves from control plants exhibited an evident senescence phenotype while similar leaves at the same positions in the transgenic plants remained green and fully expanded.

**Figure 5:**
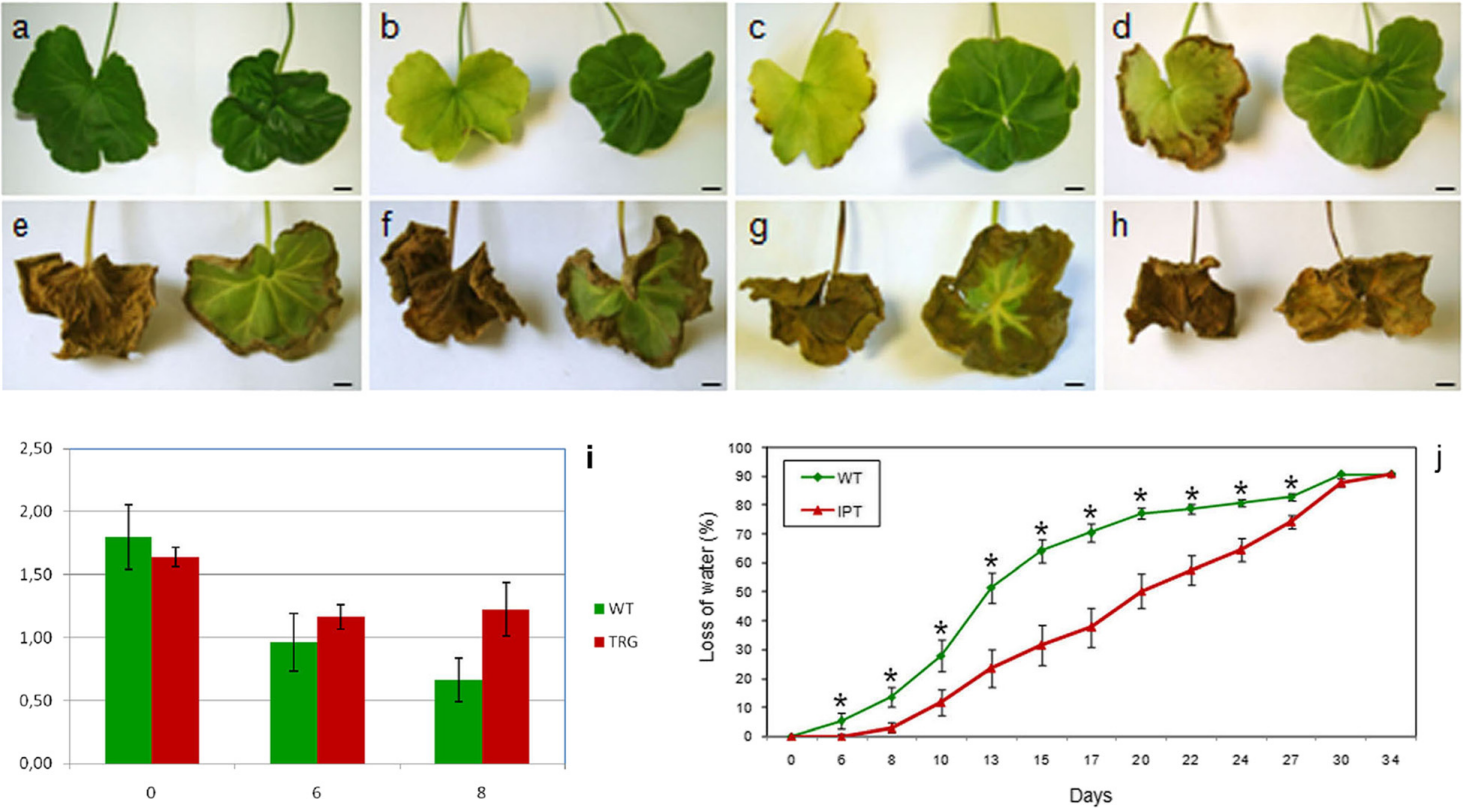
**Senescence delay in detached leaves of WT *Pelargonium zonale *and *pSAG12::ipt *transgenic plants. **Detached leaves of control (left) and *pSAG12*::*ipt* (right) plants at 0 (**a**), 6 (**b**), 8 (**c**), 17 (**d**), 22 (**e**), 24 (**f**), 27 (**g**) and 34 (**h**) days of incubation in darkness. (**i**) Mean concentration (±SE) of chlorophyll a + b (mg/g fresh weight) from detached leaves of control (WT) and *pSAG12::ipt *(TRG) plants at 0, 6 and 8 days of incubation in darkness. (**j**) Senescence delay of detached leaves from *pSAG12*::*ipt *plants. Fresh weight changes in detached leaves of WT *P. zonale *and a transgenic line carrying the *pSAG12:*:*ipt *chimaeric gene over the time course analyzed. Data are the means of sixteen leaves ± SE. Bars: 1 cm.

To better characterize and determine the delay of senescence in the transgenic plants, young and healthy leaves of similar age from both transgenic and control plants were detached and their petioles were placed in glass tubes with water at 28°C in darkness. The analysis of these leaves over time showed that leaves from the *pSAG12::ipt* transgenic plants remained green during more time than the controls. In Figure 5, we show a comparison of detached leaves from the control (left) and *pSAG12::ipt* (right) plants at 0 (a), 6 (b), 8 (c), 17 (d), 22 (e), 24 (f), 27 (g) and 34 (h) days of incubation in darkness. While the WT leaves exhibited evident symptoms of chlorophyll degradation after 6 days of incubation in darkness, the transgenic leaves exhibited similar symptoms after 22 days of incubation, indicating a delay in the senescence process. Likewise, necrotic symptoms appeared earlier in the WT leaves (~ 8 days) than in the transgenic ones (~ 20 days). Chlorophyll quantification assays in the detached leaves at 0, 6 and 8 days corroborated these observations (Figure 5i). For instance, the decline in chlorophyll (a + b) content after 8 days of incubation in darkness of WT and *pSAG12::ipt* leaves was 63.0% and 25.2% respectively, corroborating the delay in the senescence process in the transgenic plants. Moreover, water loss during the time course was lower in leaves from transgenic plants than from WT ones (Figure 5j). The differences begin to be significant after 6 days of incubation in darkness. After 15 days there was a 65% of loss of fresh weight in the WT leaves, while in the transgenic leaves the loss of weight was only a 32%. After 22 days the loss of fresh weight in the WT leaves reached the 80% of their initial weight, while the loss of water in the transgenic leaves was the 65% of their initial weight. After 30 days, the values of WT and transgenic leaves were similar. These data reinforce the idea that the chimaeric construct *pSAG12::ipt* could be useful in *Pelargonium* spp. to delay the senescence process and to produce long-lived plants.

### Early anther ablation in P. zonale transgenic plants results in efficient male sterility

**Figure 6:**
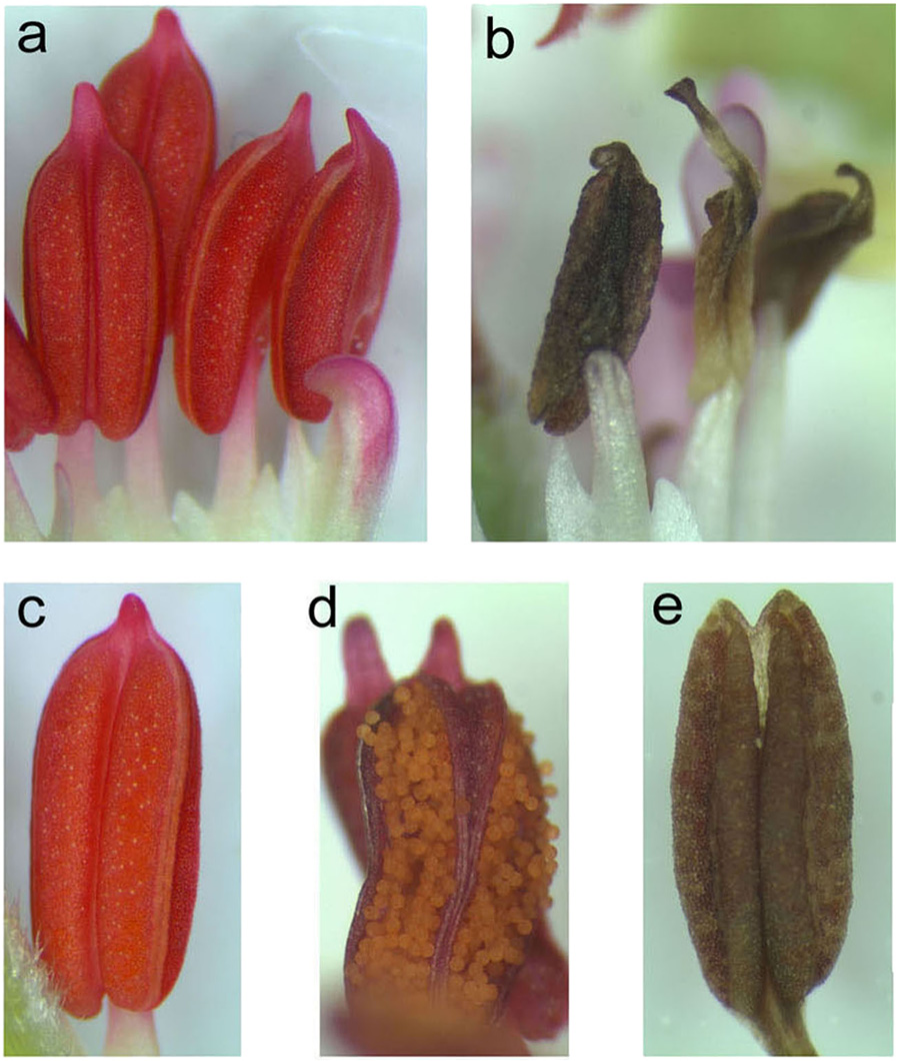
**Engineered anther ablation in the *Pelargonium zonale PsEND1::barnase *plants. **(**a**). In WT anthers from flowers one day prior to anthesis, the locules were fully developed, showing the normal shape. (**b**). Transgenic *PsEND1::barnase *anthers showing collapsed structures at the end of a short filament in the place of a normal four-lobed anther with a fully expanded filament (**c**). (**d)** Viable pollen grains in a WT anther which are visible during anthesis. (**e**), No pollen grains were observed in the modified anther structures developed instead of normal anthers in the lines carrying the male-sterility construct. The locules of sterile anthers from flowers 3–4 days prior to anthesis were narrow and unexpanded. In most cases, the undeveloped anther, composed of the ablated anther tissues, becomes a necrotic structure that never dehisces.

Transgenic *P. zonale PsEND1::barnase* plants showed normal vegetative development and flowering. However, anthers from transgenic lines carrying the chimaeric *PsEND1::barnase* gene construct showed dramatic differences in development when compared with non-transformed WT anthers. Anthers at different stages of development were examined by light microscopy. In WT anthers from flowers one day prior to anthesis (Figure 6a), the locules were fully developed, showing the normal shape (Figure 6c) and containing viable pollen grains which are visible during anthesis (Figure 6d), whereas the transgenic flowers showed collapsed structures at the end of a short filament in the place of a normal four-lobed anther (Figure 6b) with a fully expanded filament.

No pollen grains were observed in the modified anther structures which developed instead of normal anthers in the lines carrying the male sterility construct. The locules of sterile anthers were narrow and unexpanded in flowers 3–4 days prior to anthesis. In most cases, the undeveloped anther becomes a necrotic structure, composed of ablated anther tissues, that never dehisces (Figure 6e). The cross-pollination of the male-sterile plants with pollen from wild-type plants usually produced normal fruits and seeds, indicating that female fertility was not affected. In some transgenic lines, we detected a small reduction in seed production (data not shown), these lines were discarded.

## Discussion

Classical breeding programmes applied to produce new or improved varieties of floricultural species resulted in a range of cultivars with excellent traits, such as colour, shape, fragrance, vase life in cut-flower species, rooting potential or overall plant morphology. However, some of these aims have not yet been achieved in many ornamental species. Gene transfer by means of *Agrobacterium tumefaciens* enables the introduction of new genes/traits from unrelated species and would be a helpful tool in pelargonium breeding.

Efficient regeneration protocols that could be applicable to a broad spectrum of different genotypes are a prerequisite for developing a transformation system for a plant species or genotype [1]. We have developed a simple and reliable *in vitro* regeneration protocol for the genetic transformation of *Pelargonium spp.* The two *Pelargonium* genotypes used in the present study showed different regeneration ability. Using the same culture medium, regeneration was carried out via somatic embryogenesis in *P. peltatum* and via organogenesis in *P. zonale*. Interestingly, the percentage of regenerating explants obtained from both genotypes was similar (90% in *P. peltatum* and 80% in *P. zonale*) and also the transformation efficiency (2-3%).

We have also evaluated the use of the *gfp* gene as an *in vivo* selectable marker in Pelargonium. It has been reported that g*fp* expression in transformed cells is useful to select transformation events in early stages, so that antibiotic selection is not needed [47,54-61]. In the case of *Pelargonium spp*., the use of *gfp* as a selectable *in vivo* marker gene is restricted to identify transformation events, because at late developmental stages it becomes undetectable due to the presence of chlorophyll in the green tissues (leaves). In adult plants, *gfp* expression is only detectable in non green tissues like roots, petals or anther filaments.

Using this transformation protocol, we introduced two new traits in *P. zonale* cv. 370, one to produce long-life plants by inducing the *ipt* gene during plant senescence and the other to produce male sterile plants without pollen. During the initial transformation assays we used the *gfp* reporter gene to *in vivo* identify the transformations events and to evaluate transformation efficiency in the regenerating plantlets. The *gfp* gene has been used to successfully transform sugar cane, tobacco, maize, lettuce [54], walnut [55], citrus [56], peach [57], potato [58], pear [59], carrot [60] and other species [61]. In *P. zonale*, we have observed *gfp* expression along the regeneration process, but, at times longer than three weeks after inoculation, we could not correlate explants that regenerated transgenic plants with green fluorescence. Low levels of *gfp* fluorescence coincided in time with increased content of chlorophyll and the red autofluorescence of chlorophyll interferes with the *gfp* green fluorescence [62]. This interference could also be caused by pigments that are opaque to exciting UV or blue light [61]. Some authors state that transformation efficiencies based on resistance to a selective marker are probably underestimating the actual rate of regenerated transgenic plants [63]. Therefore PCR analysis has been proposed to identify transgenic plants in addition to the use of selectable or visual screening markers [63]. In the present work, the *gfp* gene was useful to confirm genetic transformation of *P. zonale in vivo.* All transgenic plants showed green fluorescence in nearly all tissues analyzed but there were large differences in green fluorescence between organs and tissues, depending on the chlorophyll content of each one. PCR analysis corroborated the presence of transgenes in the regenerated plantlets.

In genetic transformation experiments, the analyses generally focus on the molecular characterization of the transgenic plants but the ploidy level of the transgenic material is checked in relatively few cases. The confirmation of the ploidy level in transgenic material is particularly important during the selection of transgenic lines. Our results indicate that leaf tissues of the *Pelargonium* cultivars used here have a diploid number of chromosomes and regeneration from these explants leads to plants with the same ploidy level as adult material of the original cultivar.

Transgenic *pSAG12::ipt* plants showed delayed leaf senescence, which was more evident at the basal leaves. They also showed increased branching and reduced internodal length when compared with non transformed plants. In addition, the transgenic *pSAG12::ipt* plants displayed a more compact architecture than the WT plants. Other interesting phenotypic difference among the WT and the transgenic *pSAG12::ipt* plants was the reduction of transgenic leaf size. The plant architecture was compact in the transgenic plants, including tight inflorescences and flowers. In some *pSAG12::ipt* inflorescences, flowers coexisted with new vegetative structures which are produced at the same time as flowers or new inflorescences. This occasional phenomenon might be due to a change in the determination of the floral meristem leading to inflorescence reversion and new vegetative organs instead of flowers in the inflorescences.

Inflorescence or flower reversion may occur when the level of the floral signal is insufficient for the completion of flower development and the suppression of indeterminacy [64]. Flower and inflorescence reversion involve a switch from floral development back to vegetative development, thus rendering flowering a phase in an ongoing growth pattern rather than a terminal act of the meristem. Although it can be considered an unusual event, it is linked to environmental conditions and is most often a response to conditions opposite to those that induce flowering. A clear-cut reversion to leaf production has been described in *Impatiens balsamina*[65]. In *I. balsamina*, a leaf-derived signal is critical for completion of flowering and can be considered to be the basis of a plant-wide floral commitment that is achieved without accompanying meristem autonomy. It has been proposed that cytokinins can be involved in floral induction as the leaf-generated signal that produces completion of the flowering process making it irreversible [66,67]. These cytokinin fluxes during floral induction in LD plants could be altered in the *pSAG12::ipt* transgenic plants during senescence due to the continued production of cytokinin and this fact could be influencing the reversion process in some inflorescences. To elucidate if there was a correlation between the inflorescence reversion phenotype and the expression level of the exogenous gene in these plants, we carried out real-time RT-PCR experiments. Our results indicated that the expression level of the transgene is higher in those transgenic lines showing inflorescence reversion. However, this phenotype could be considered as an undesirable collateral effect from a commercial point of view and transgenic lines showing occasional inflorescence reversion were discarded.

All the *pSAG12::ipt P. zonale* plants cultivated in the greenhouse exhibited delayed senescence when compared with WT control plants, especially in the basal leaves. A high number of adult leaves of control plants exhibited an evident senescence phenotype while similar leaves at the same positions in the transgenic plants remained green and fully expanded. To better characterize and determine the delay of senescence in the transgenic plants, young and healthy leaves of similar age from both transgenic and control plants were detached and their petioles were placed in glass tubes with water at 28°C in darkness. The analysis of these leaves over time showed that leaves from the *pSAG12::ipt* transgenic plants remained green longer than the controls. While the WT leaves exhibited evident symptoms of chlorophyll degradation after 6 days of incubation in the darkness, the transgenic leaves exhibited similar symptoms after 22 days of incubation, indicating a delay in the senescence process. Likewise, necrotic symptoms appeared early in the WT leaves than in the transgenic ones. Quantification of chlorophyll content of detached WT and *pSAG12::ipt* leaves indicated that the decline of chlorophyll was higher in the WT leaves when compared with the transgenic ones. Moreover, the loss of water in leaves from transgenic plants was minor over the time course analyzed. These data reinforce the idea that the chimaeric *pSAG12::ipt* construct could be useful in *Pelargonium* spp. to delay the senescence process and to produce long-lived plants.

We have obtained engineered male sterile plants of *P. zonale* to prevent undesirable lateral gene flow of the introduced transgenes to related species. The *PsEND1* promoter specifically directed expression of the *barnase* gene to different anther tissues involved in anther architecture (epidermis, endothecium, middle layer, connective tissue). Expression of the *barnase* gene under control of this promoter caused specific ablation of these tissues at early stages of anther development in the transgenic plants. We readily observed small structures that developed instead of normal anthers in the third floral whorl of transgenic flowers, which displayed premature senescence and collapse of the pollen sacs, microspores and tapetum and a lack of pollen at anthesis. Ablation of the structural anther tissues also produces the improper formation of the tapetum tissue and the subsequent degeneration of the microspores is accompanied by a change in anther wall thickness, by a size reduction and by a change in the epidermal cell types [38]. Since this phenotype is unlikely to be due to expression of barnase in structural tissues, it is likely to be an indirect effect of the loss of the tapetum and microspore cells.

No pollen grains were observed in any section of the ablated anthers from the male sterile plants, indicating that barnase effectively ablates specific cell lines that will form the structural tissues of the anther, preventing pollen development. Transgenic anthers appeared to show effects of barnase expression at every stage examined. This is likely due to the developmentally earlier expression of the *PsEND1* promoter. The anther filaments of the transgenic plants were shorter than WT filaments. The formation of short filaments is commonly associated with male sterility or reduced fertility [43,68-70]. These observations reinforce our previous results in other crop species using the same chimaeric construct [38].

Due to the extremely toxic nature of barnase, other researchers have reported a general lack of vigour and a decline in plant growth in transgenic plants carrying the *barnase* gene [71-73]. The lack of significant effects on growth characteristics is important to know when considering the use of barnase for male sterility in landscaping plants. To prevent the possible effects of ectopic *barnase* expression, Gardner et al. [40] proposed the introduction of the male and/or female sterility genes in combination with a gene protecting against inappropriate *barnase* expression (enhanced *35S::barstar*). In all the lines of transgenic *P. zonale* plants expressing *barnase* under control of the *PsEND1* promoter, we did not observe differences with respect to wild type plants in vegetative growth, flowering time or inflorescence number. Morphological analysis of the transgenic plants showed that, under greenhouse conditions, the expression of the *PsEND1::barnase* construct does not significantly affect the vegetative and floral development, thus confirming the anther specificity of the *PsEND1* promoter region previously observed by means of the GUS expression studies in different dicots and monocots [38,39,41,42]. The potential biotechnological applications of the *PsEND1* promoter largely depend on both its spatial and temporal expression pattern, since the ectopic expression of the cytotoxic agent would damage other plant tissues and organs, decreasing the agronomic value of hybrid plants.

Expression of the *barnase* gene in ornamental plants under control of the anther-specific *PsEND1* promoter may be used to create efficient male sterile versions of existing popular cultivars without adversely affecting their respective phenotypes. Therefore, this technology would be especially useful to produce environmentally friendly transgenic plants carrying new traits by preventing gene flow between the genetically modified ornamentals.

## Conclusions

We have developed a simple and reliable *in vitro* regeneration protocol for the genetic transformation of *Pelargonium spp*. By using this methodology, we introduced two new traits in *P. zonale* cv. 370, one to produce long-life plants and the other to produce male sterility. The resulting phenotypes would be of interest both for consumers and producers.

The chimaeric *pSAG12::ipt* construct may be useful in *Pelargonium spp.* to delay the senescence process and to produce long-lived plants, which could have commercial interest. Transgenic *pSAG12::ipt* plants showed delayed leaf senescence, increased branching and reduced internodal length as compared to non-transformed plants. Moreover, the transgenic *pSAG12::ipt* plants showed a more compact architecture than the WT. In some *pSAG12::ipt* inflorescences, flowers coexist with new vegetative structures which are produced at the same time as flowers or new inflorescences. This occasional phenomenon might be due to a change in the determination of the floral meristem leading to inflorescence reversion as a collateral effect of the increased expression levels of the transgene in some transgenic lines. The lines showing inflorescence reversion were discarded because this effect is undesirable from a commercial point of view.

The *PsEND1* promoter specifically directed expression of the *barnase* gene to different anther tissues involved in anther architecture. Expression of the *barnase* gene under control of this promoter caused specific ablation of these tissues, which become necrotic at early stages of anther development in the transgenic pelargonium plants. No pollen grains were observed in the ablated anthers from the male-sterile plants, indicating that barnase effectively destroys specific cell lines that form the structural tissues of the anther, thus preventing pollen development. The use of engineered male sterility would be especially useful to eliminate pollen allergens and to produce environmentally friendly transgenic plants carrying new traits by preventing gene flow between the genetically modified ornamentals and related plant species.

## Abbreviations

AS: Acetosyringone; BA: 6-benzylaminopurine; CEF: Cefotaxime; *Gfp*: Green fluorescent protein gene; *Ipt*: Isopentenyl phosphotransferase gene; IAA: Indole-3-acetic acid; KAN: Kanamycin sulphate; KIN: Kinetin; LB: Luria Bertani medium; MES: 2-(*N*-morpholino)ethane sulfonic acid; MS: Murashige and Skoog medium; NAA: α-naphthalene acetic acid; *nptII*: Neomycin phosphotransferase gene; PCR: Polymerase chain reaction; TDZ: Thidiazuron; TIM: Timentin (ticarcillin/clavulanic acid); *uidA*: β-glucuronidase gene.

## Competing interests

In the past five years we have received funding from the Spanish Ministry of Science and Innovation (MICINN) and the article-processing charge will be pay with funds from two granted projects. The authors received salaries from two different institutions: The Polytechnic University of Valencia (UPV) or the High Spanish Council of Scientific Research (CSIC). We are not currently applying for a patent related with the content of this manuscript. All the mentioned organisms/institutions do not gain or lose financially from the publication of this manuscript either now or in the future.

## Authors’ contributions

BG-S and BP performed the transformation experiments of *P. zonale*, chlorophyll quantification and loss of water experiments, both authors contributed equally to this paper. TA contributed to the production of *in vitro* regenerated and greenhouse *P. zonale* plants. AA performed the ploidy level analyses of transgenic lines. MB and VM designed the experiments related to the production of *pSAG12::ipt* transgenic plants. ER performed the molecular analyses (PCR and real-time RT-PCR) of transgenic plants. JPB and LAC designed the experiments related to the production of male sterile *PsEND1::barnase* transgenic plants. All authors read and approved the final version of the manuscript.
